# Threshold Learning Dynamics in Social Networks

**DOI:** 10.1371/journal.pone.0020207

**Published:** 2011-05-27

**Authors:** Juan Carlos González-Avella, Victor M. Eguíluz, Matteo Marsili, Fernado Vega-Redondo, Maxi San Miguel

**Affiliations:** 1 Instituto de Física Interdisciplinar y Sistemas Complejos IFISC (CSIC-UIB), Palma de Mallorca, Spain; 2 The Abdus Salam International Centre for Theoretical Physics, Trieste, Italy; 3 European University Institute, Florence, Italy; 4 Instituto Valenciano de Investigaciones Económicas, Valencia, Spain; University of Zaragoza, Spain

## Abstract

Social learning is defined as the ability of a population to aggregate information, a process which must crucially depend on the mechanisms of social interaction. Consumers choosing which product to buy, or voters deciding which option to take with respect to an important issue, typically confront external signals to the information gathered from their contacts. Economic models typically predict that correct social learning occurs in large populations unless some individuals display unbounded influence. We challenge this conclusion by showing that an intuitive threshold process of individual adjustment does not always lead to such social learning. We find, specifically, that three generic regimes exist separated by sharp discontinuous transitions. And only in one of them, where the threshold is within a suitable intermediate range, the population learns the correct information. In the other two, where the threshold is either too high or too low, the system either freezes or enters into persistent flux, respectively. These regimes are generally observed in different social networks (both complex or regular), but limited interaction is found to promote correct learning by enlarging the parameter region where it occurs.

## Introduction

Social learning has been a topic of central concern in economics during the last decades [1], as it is central to a wide range of socio-economic phenomena. Consumers who want to choose among a given set of available products may seek the opinion of people they trust, in addition to the information they gather from prices and/or advertisement. And voters who have to decide what candidate to support in an election, or citizens who have to take a stand on some issue of social relevance may rely on their contacts to form their opinion. Ultimately, whether our societies take the right course of action on any given issue (e.g. on climate change) will hinge upon our ability to aggregate individual information that is largely disperse. Thus, in particular, it must depend on the information diffusion mechanism by which agents learn from each other, and therefore on the underlying social network in which they are embedded. The significance of the conceptual challenges raised by these issues is made even more compelling by the booming advance in Information and Communication Technologies, with its impact on the patterns of influence and communication, and on the way and speed in which we communicate.

These key issues have attracted the interest of researchers in several fields. For example, the celebrated “voter model” [2], [3] is a prototype of those simple mechanistic models that are very parsimonious in the description of individual behavior but allow for a full characterization of the collective behavior induced. The voter model embodies a situation where each agent switches to the opinion/state held by one of the randomly selected neighbors at some given rate, and raises the question of whether the population is able to reach consensus, i.e. a situation where all agents display the same state. The literature on consensus formation, as reviewed e.g. in Refs. [4], [5], has focused, in particular, on the role played by the structure of the underlying network in shaping the asymptotic behavior. One of the main insights is that the higher the effective dimensionality of the network, the harder it is to obtain conformity [6], [7]. Consensus formation in social systems is closely related to the phenomenon of social learning. Indeed, the latter can be regarded as a particular case of the former, when consensus is reached on some “true” (or objective) state of the world, for example, given by an external signal [8], [9] impinging on the social dynamics.

At the opposite end of the spectrum, economists have stressed the *micro-motives* that underlie individual behavior and the assumption of rationality. They have also emphasized the importance of going beyond models of global interaction and/or bilateral random matching, accounting for some local structure (modeled as a social network) in the pattern of influence or communication among agents. This literature (see Ref. [10] for an early survey) has considered a number of quite different scenarios, ranging from those where agents just gather and refine information [11]–[13] to contexts where, in addition, there is genuine strategic interaction among agents [14]. Despite the wide range of specific models considered, the literature largely conveys a striking conclusion: full social conformity is attained (although not necessarily correct learning), irrespectively of the network architecture. On the other hand, to attain correct learning, one must require not only that the population be large but, in the limit, that no individual retain too much influence [14], [15].

The model studied in this paper displays some similarities to, as well as crucial differences with, those outlined above. To fix ideas, the model could be regarded as reflecting a situation where, despite the fact that new information keeps arriving throughout, the consequences of any decision can only be observed in the future. As a more concrete example, this could apply to the performance of a political candidate, the health consequences of consuming a particular good, or the severity of the problem of climate change, on all of which a flow of fresh information may be generated that is largely independent of agents' evolving position on the issue. So, as in Ref. [9], the agents receive an external signal; however, the signal is noisy and it is confronted with the behavior displayed by neighbors. As in Refs. [12], [14], while the agents make and adjust their choices, they keep receiving noisy signals on what is the best action. In contrast, however, these signals are not associated to experimentation. In this respect, we share with Ref. [13], [15] the assumption that agents' arrival of information is not tailored to current choices.

The problem, of course, would become trivially uninteresting if agents either had unbounded memory or stored information that is a sufficient statistic of the whole past (e.g. updated beliefs in a Bayesian setup). In this case, agents could eventually learn the best action by relying on their own information alone. Therefore we make the stylized assumption that the particular action currently adopted by each individual is the only “trace” she (and others) keep of her past experience. Thus her ensuing behavior can only be affected by the signal she receives and the range of behavior she observes (i.e., her own as well as her neighbors'). Under these conditions, it is natural to posit that if an agent receives a signal that suggests changing her current action, she will look for evidence supporting this change in the behavior she observes on the part of her neighbors. And then, only if a sufficiently large fraction of these are adopting the alternative action, she will undertake the change. This, indeed, is the specific formulation of individual learning studied in the present paper, which is in the spirit of the many threshold models studied in the literature, such as Refs. [16]–[20]. This formulation can be conceived as the outcome of a situation where agents use Bayes rule to update their actions rather than their beliefs, and have no memory of the past (see Methods). The confidence they have on their current choice (as embodied by their subjective probability that they are right), as well as the corresponding confidence they attach to the signals and and neighbors' choices are taken as fixed parameters. Then, essentially, what is required for an agent to follow a threshold rule as described is that she attaches a high confidence to both her current choice and her current signal. For, under these conditions, when the signal and the choice point in opposite directions – and only then – any change of behavior will hinge upon sufficient strong support for it provided by the neighbors' actions.

In the setup outlined, it is intuitive that the “acceptance threshold” that agents require to abandon the status quo should play a key role in the overall dynamics. And, indeed, we find that its effect is very sharp. First, note the obvious fact that if the threshold is either very high or very low, social learning (or even behavioral convergence) cannot possibly occur. For, in the first case (a very high threshold), the initial social configuration must remain frozen, while in the second case (a very low threshold), the social process would enter into a state of persistent flux where agents keep changing their actions. In both of these polar situations, therefore, the fraction of agents choosing the good action would center around the probability 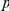 with which the signal favors that action.

Outside these two polar situations, there is always an intermediate region where social learning does occur. Within this region, learning emerges abruptly: there are upper and lower bounds (dependent on 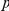) such that, if the threshold lies within these bounds, *all* agents learn to play the good action while *no* learning *at all* occurs if the threshold is outside that range. Thus the three aforementioned regions are separated by sharp boundaries. A similar abruptness in learning arises as one considers changes in 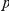. In this case, there is a lower bound on 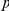 (which depends on the threshold) such that, again, we have a binary situation (i.e., no learning or a complete one) if the informativeness of the signal is respectively below or above that bound. In a sense, these stark conclusions highlight the importance of the social dimension in the learning process. They show that, when matters/parameters are “right,” the process of social learning builds upon itself to produce the sharp changes just outlined. The situation is reminiscent of the “tipping-point” behavior that has been widely studied for epidemic phenomena and which, as it is well understood by now, often characterize as well the transitions of complex dynamic systems.

As it turns out, this same qualitative behavior is encountered in a wide variety of different network contexts. To understand the essential features at work, we start our analysis by studying the simple case of a complete graph, where every agent is linked to any other agent. This context allows one to get a clear theoretical grasp of the phenomenon. In particular, it allows us to characterize analytically the three different regimes of social learning indicated: correct learning, frozen behavior, or persistent flux. We then show that this characterization also provides a good qualitative description of the situation when the interaction among agents is mediated via a sparse complex network. We consider, in particular, three paradigmatic classes of networks: regular two-dimensional lattices, Poisson random networks, and Barabási-Albert scale free networks. For all these cases, we conduct numerical simulations and find a pattern analogous to the one observed for the complete graph. The interesting additional observation is that local interaction *enlarges* (in contrast to global interaction) the region where social learning occurs. In fact, this positive effect is mitigated as the average degree of the network grows, suggesting a positive role for relatively limited/local connectivity in furthering social learning.

### The model

There is large population of agents, 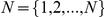, placed on a given undirected network 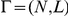, where we write 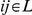 if there is link between nodes 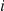 and 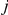 in 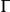. Let update step 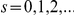 be indexed discretely. Each agent 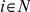 displays, at any step 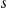, one of two alternative actions 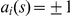, which are not equivalent. One of them, say action 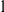, induces a higher (expected) payoff, but the agents do not know this.

At each step 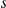, one randomly chosen agent 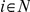 receives a signal on the relative payoff of the two actions. This signal, which is independent across time and agents, is only partially informative. Specifically, it provides the correct information (i.e., “action 1 is best”) with probability 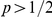, while it delivers the opposite information with the complementary probability 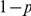.

If agent 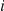
's previous action 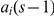 does not coincide with the action 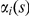 suggested as best, she considers whether changing to the latter. We assume that she chooses 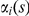 (thus making 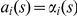) if, and only if, the fraction of neighbors in 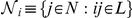 who chose 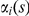 at 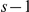 exceeds a certain threshold. Let this (common) threshold be denoted by 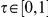.

At the start of the dynamic process, each agent receives one signal 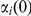 and adopts the corresponding action. In other words, the initial condition for the process is one where each agent, independently, holds action 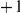 with probability 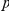 or action 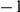 with probability 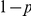.

The central question posed in the paper can now be precisely formulated:

What is the relationship between 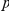 (the quality of the signal) and 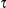 (the threshold for action change) that underlies the spread and consolidation of action 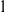
?

This is the question addressed in what follows, in a range of different setups and relying on a variety of methodologies.

## Results

### Global interaction for infinite populations

Let us consider the case where interaction is global: for each pair of agents 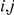 we have that 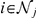 and 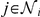. Let 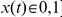 stand for the fraction of agents choosing action 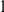 at time 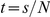. Setting the time in this way, for each agent the average number of updates per unit of time is 1, irrespective of system size 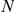. In the limit of infinite population size (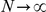), the dynamics is given by:
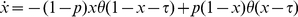
(1)


where 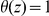 if 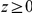 while 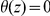 if 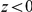. This equation is derived by considering the change 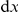 in the fraction 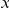 occurring in a time interval of 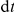 time steps (equivalently, 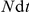 updates). For 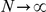, for any finite 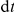, this increment converges, by the law of large numbers, to a constant given by the right hand side of Eq. (1) times 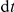. The first term accounts for the number of agents initially with the right signal (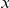) who receive the wrong signal (with probability 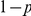) and adopt it, as the fraction of agents also adopting it is larger than the threshold (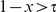). The second accounts for the opposite subprocess, whereby agents who receive the correct signal (with probability 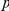) switch to the correct action when the population supports it (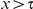).

We assume that, at time 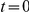, each agent receives a signal 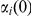 and adopts the corresponding action 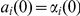. Hence the initial condition for the dynamics above is 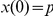.

It is useful to divide the analysis into two cases:

**Case I.** 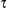 *>*1*/*2

In this case, it is straightforward to check that
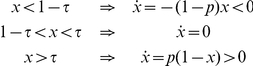



So, it follows that correct social learning, in which the whole population adopts the action privileged by the signal (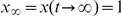), occurs iff 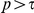.

**Case II.** 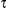 *<*1*/*2

In this case, we find:
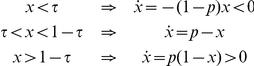



And, therefore, correct social learning occurs iff 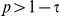.

Combining both cases, we can simply state that, in the global interaction case, correct social learning occurs iff,
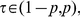
(2)


that is, the threshold 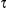 is within an intermediate region whose size grows with the probability 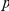, which captures the informativeness of the signal. However, there are other two phases: if 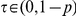, the system reaches the stationary solution 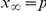; while if 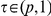, we have 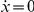 for all times, which means that the system stays in the initial condition 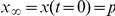.

It should be noted that the frozen solution 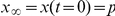 only exists rigourously for the step-wise threshold function used in our model. In a model with a blurred threshold in which 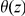 is replaced in equation (1), for example, by 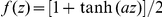, then 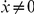 in the interval 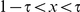, and there is complete learning for 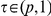. However, for reasonable large values of the parameter 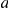 for which the threshold concept is still meaningful, the dynamical flux is so small that the asymptotic learning solution is only reached numerically on extremely long time scales. In fact, in the numerical simulations described below for discrete systems with a large population, only the frozen solution 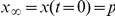 is observed for the whole duration of the simulations, even when smoothing out the threshold function.

### Numerical simulations

Now we explore whether the insights obtained from the infinite size limit of the global interaction case carry over to setups with a finite but large population, where agents are connected through a social network.

First, we consider the benchmark case of global interaction (i.e., a completely connected network). Then, we turn to the case of local interaction and focus on three paradigmatic network setups: lattice networks, Erdös-Rényi (Poisson) networks, and Barabási-Albert (scale-free) networks [21].

### Global interaction

**Figure 1:**
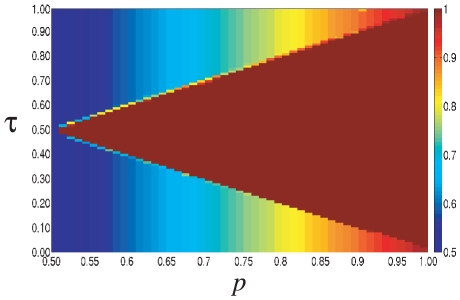
Phase diagram of the threshold model on a fully connected network. The colors represent the fraction of agents choosing action 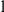 (from red, 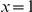 to blue, 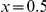). System size given by 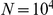 agents; averaged over 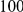 realizations.

The results obtained on the completely connected network (i.e., the network where every pair of nodes is linked) are in line with the theory presented in the previous section. The essential conclusions can be summarized through the phase diagram in the 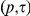-space of parameters depicted in Figure 1. There we represent the fraction of agents choosing action 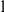 in the steady state for each parameter configuration, with the red color standing for a homogeneous situation with 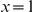 (i.e., all agents choosing action 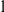) while the blue color codes for a situation where 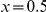 and therefore the two actions are equally present in the population. Intermediate situations appear as a continuous color grading between these two polar configurations.

**Figure 2:**
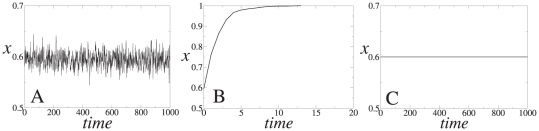
Typical realizations of the time evolution of the fraction of agents choosing action 1, 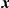, in a fully connected network of system size 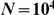 with 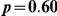, and (*A*) 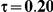; (*B*) 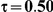; (*C*) 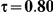.

We find that, depending on the quality of the external signal 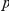 and the threshold 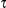, the system reaches configurations where either complete learning occurs (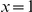) or not (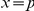). Indeed, the observed asymptotic behavior is exactly as predicted by the analysis of the previous section and it displays the following three phases:

- Phase I: 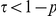. The system reaches a stationary aggregate configuration where the nodes are continuously changing their state but the average fraction of those choosing action 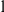 gravitates around the frequency 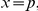 with some fluctuations (see Figure 2 *A*). The magnitude of these fluctuations decreases with system size 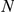.
- Phase II: 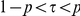. The system reaches the absorbing state 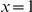 where everyone adopts action 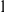. This is a situation where the whole population eventually learns that the correct choice is action 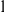 (see Figure 2 *B*).
- Phase III: 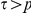. The system freezes in the initial state, so the fraction 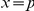 of agents choosing the correct action coincides with the fraction of those that received the corresponding signal at the start of the process (see Figure 2 *C*).

**Figure 3:**
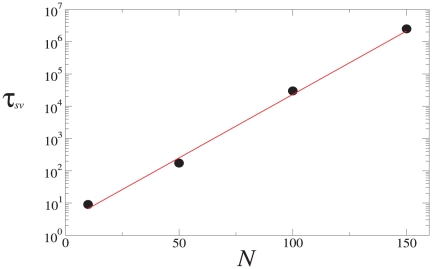
The average survival time 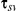 in fully connected networks for different system sizes 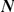 for 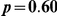 and 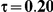. The continuous line corresponds to an exponential fit of the form 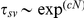, being 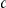 a constant; averaged over 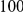 realizations.

It is worth noting that, while in Phase I the theory predicts 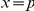, any finite-size system must eventually reach an absorbing homogenous state due to fluctuations. Thus, to understand the nature of the dynamics, we determine the average time 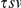 that the system requires to reach such an absorbing state. As shown in Figure 3, 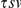 grows exponentially with 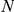. This means that 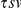 grows very fast with system size, and thus the coexistence predicted by the theory in Phase I can be regarded as a good account of the situation even when 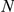 is just moderately large.

### Lattice networks

Now assume that all nodes are placed on a *regular boundariless lattice* of dimension 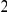, endowed with the distance function 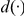 given by 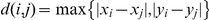. The social network is then constructed by establishing a link between every pair of agents lying at a lattice distance not larger than a pre-specified level 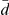. This defines the neighborhood 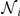 of any agent 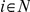, as given by 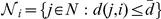. In this network, the degree (i.e. the number of neighbors) of any node 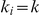 is related to 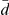; for instance, if 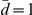 we have 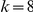.

**Figure 4:**
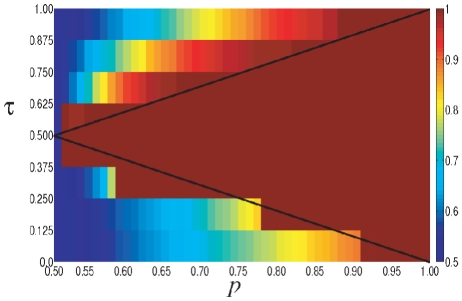
Phase diagram of the threshold model on a two-dimensional lattice with 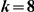 (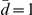). The colors represent the fraction of agents choosing action 1 (from red, 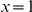, to blue, 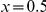). System size 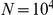; average over 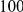 realizations.

The behavior of the system is qualitatively similar to the case of a fully connected network. Again we find three phases. In two of them, both actions coexist with respective frequencies 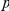 and 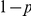 (one phase is frozen and the other continuously fluctuating), while in the other phase the whole population converges to action 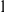. A global picture of the situation for the entire range of parameter values is shown in Figure 4, with the black diagonal lines define the boundaries of the full-convergence region under global interaction. In comparison with the situation depicted in Figure 1, we observe that the region in the 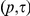-space where behavioral convergence obtains in the lattice network is broader than in the completely connected network. This indicates that restricted (or local) interaction facilitate social learning, in the sense of enlarging the range of conditions under which the behavior of the population converges to action 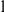.

**Figure 5:**
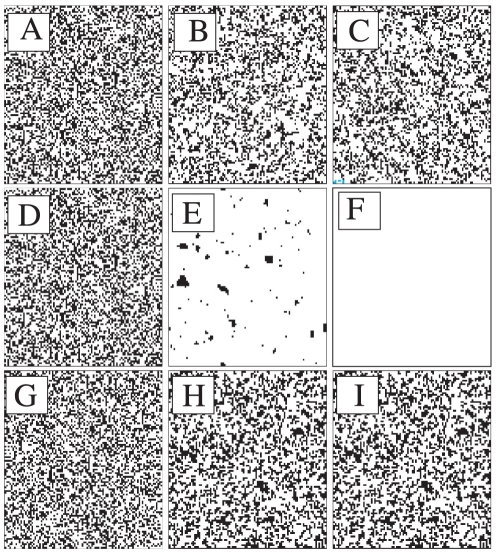
Time evolution of the threshold model on a two-dimensional lattice with 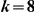 for different values of 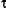 and 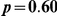. Panels (*A*–*C*): 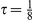 and time steps (*A*) 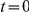, (*B*) 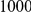 and (*C*) 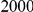. Panels (*D*–*F*): 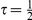 and time steps (*D*) 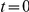, (*E*) 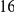 and (*F*) 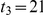. Panels (*G*–*I*): 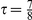 and time steps (*G*) 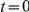, (*H*) 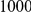 and (*I*) 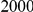. Black color represents an agent using action 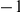, while white color represents action 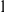. The system size is 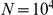.

As a useful complement to the previous discussion, Figure 5 illustrates the evolution of the spatial configuration for a typical simulation of the model in a lattice network, with different values of 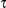 and 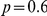. Panels 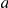, 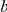 and 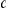 show the configurations of the system for a low value of 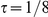 at three different time steps: 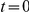, 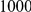 and 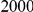 respectively. The evolution of the system displays a configuration analogous to the initial condition, both actions coexisting and evenly spreading throughout the network. This is a situation that leads to dynamics of the sort encountered in *Phase* I above. In contrast, Panels 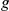, 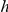 and 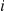 correspond to a context with a high 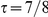, which induces the same performance as in *Phase* III. It is worth

emphasizing that although Panels 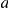, 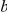 and 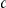 display a similar spatial pattern, they reflect very different dynamics, i.e., continuous turnover in the first case, while static (frozen initial conditions) in the second case. Finally, Panels 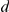, 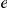 and 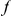 illustrate the dynamics for an intermediate value of 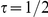, which leads to a behavior of the kind displayed in *Phase* II. Specifically, these panels show that, as the system moves across the three time steps: 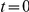, 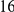 and 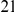, the system evolves, very quickly, toward a state where all agents converge to action 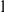.

### Erdös-Rényi and scale-free networks

A lattice network is the simplest possible context where local interaction can be studied. It is, in particular, a regular network where every agent faces exactly symmetric conditions. It is therefore interesting to explore whether any deviation from this rigid framework can affect our former conclusions. This we do here by focusing on two of the canonical models studied in the network literature: the early model of Erdös and Rényi (ER) [22] and the more recent scale-free model introduced by Barabási and Albert (BA) [23]. Both of them abandon the regularity displayed by the lattice network and contemplate a non-degenerate distribution of node degrees.

The ER random graph is characterized by a parameter 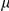, which is the connection probability of agents. It is assumed, specifically, that each possible link is established in a stochastically independent manner with probability 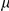. Consequently, for any given node, its degree distribution 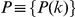 determining the probability that its degree is 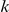 is Binomial, i.e., 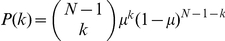, with an expected degree given by 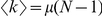. In the simulations reported below, we have focused on networks with 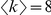 and 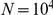.

On the other hand, to build a BA network, we follow the procedure described in Ref. [23]. At each step, a new node is added to the network and establishes 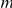 links to existing nodes. The newcomer selects its neighbors randomly, with the probability of attaching to each of the existing nodes being proportional to their degree 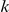. It is well known that this procedure generates networks whose degree distribution follows a power law of the form 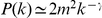, with 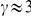. For our simulations, we have constructed BA networks using this procedure and a value of 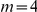, leading to an average degree 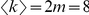.

The networks are constructed, therefore, so that they have the same average degree in both the ER and BA contexts. It is important to emphasize, however, that the degree distributions obtained in each case are markedly different. While in the former case, the degree distribution induces an exponentially decaying probability for high-degree nodes, in the latter case it leads to “fat tails”, i.e. associates significant probability to high-degree nodes.

**Figure 6:**
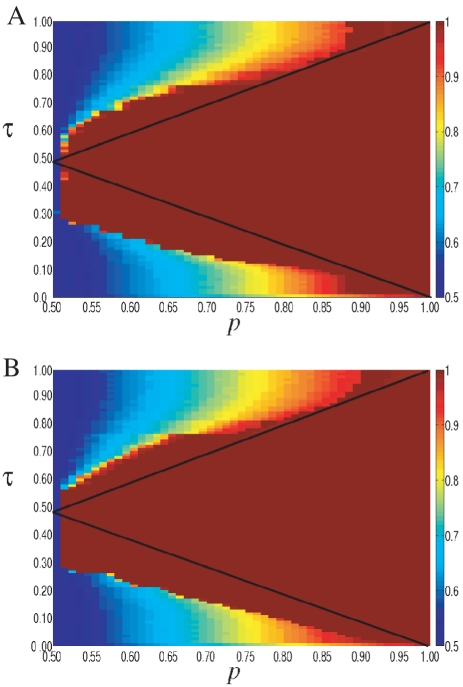
Phase diagram of the threshold model in a (*A*) ER network and in a (*B*) scale-free network with average degree 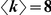. The colors represent the fraction of agents choosing action 1 (from red, 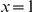, to blue 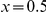). System size 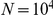, average over 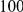 realizations.

The results are illustrated in Figure 6. For the two alternative network topologies, the system displays qualitatively the same behavior found in the lattice network. That is, there are three distinct phases yielding distinct kinds of dynamic performance: convergence to action 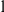, frozen behavior, and persistent turnover. However, it is interesting to note that, compared with the case of global interaction, the convergence region (which we labeled as Phase II before) is significantly larger. This suggests that local (i.e. limited) connectivity facilitates social learning.

Why does limited connectivity extend the learning region? Intuitively, the reason is that it enhances the positive role in learning played by random fluctuations. Such fluctuations are neglected, by construction, in the mean-field approximation and are also minimized when the whole population interacts globally. But, when interaction is local, those fluctuations will tend to de-stabilize the situation in both the constant flux and in the frozen phases – at first, locally, but then also globally.

To gain a more refined understanding of this issue, let us try to assess the effect of local interaction on the likelihood that, at some random initial conditions, any given node faces a set of neighbors who favors a change of actions. This, of course, is just equal to the probability that the fraction of neighbors who display opposite behavior is higher than 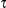, the required threshold for change. Thus, more generally, we want to focus on the conditional distribution densities 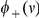 and 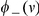 that specify, for an agent displaying actions 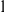 and 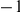 respectively, the probability density of finding a fraction 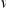 of neighbors who adopt actions 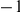 and 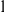, respectively. Of course, these distributions must depend on the degree distribution of the network and, in particular, on its average degree. Specifically, when the average degree of the network is large relative to population size (thus we approximate a situation of global interaction) those distributions must be highly concentrated around 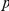 and 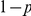 respectively. Instead, under lower connectivity (and genuine local interaction), the distributions 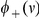 and 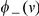 will tend to be quite disperse.

Next, let us understand what are the implications of each situation. In the first case, when the connectivity is high, the situation is essentially captured by a mean-field approximation, and thus the induced dynamics must be well described by the global interaction case (in particular, as it concerns the size of the convergence region). In contrast, when the connectivity is low and the distributions 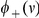 and 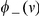 are disperse, a significant deviation from the mean-field theory is introduced. In fact, the nature of this deviation is different depending on the level of the threshold 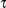. If it is low, and thus action turnover high, it mitigates such turnover by increasing the probability that the fraction of neighbors with opposite behavior lie below 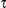. Instead, if 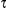 is high and action change is difficult, it renders it easier by increasing the probability that the fraction of neighbors with opposite behavior lies above 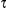. Thus, in both cases it works against the forces that hamper social learning and thus improves the chances that it occurs.

**Figure 7:**
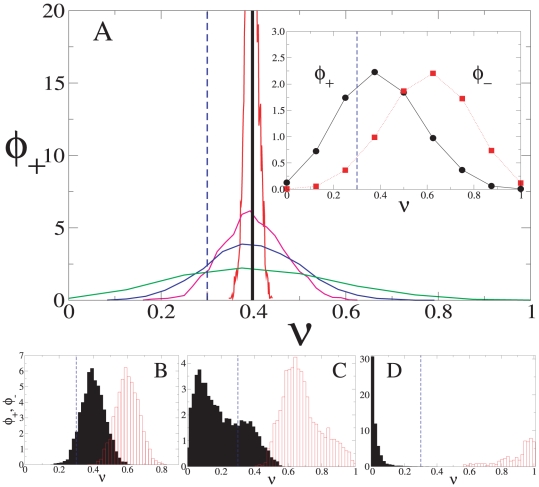
Influence of local connectivity in social learning (*A*). The initial probability density 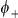 that a node using action 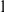 has a fraction 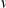 of neighbor nodes with action 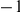, computed on a two-dimensional lattice for 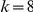, 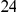, 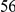, 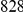 and a completely connected network (from the broadest to the narrowest probability density distribution). [*Inset*: 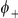 (black, continuous) and 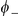 (red, dotted) for 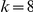.] Time evolution of the probability densities 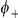 (black) and 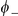 (red) in a two-dimensional lattice with 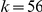 for (*B*) 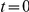, (*C*) 5 and (*D*) 10. For all panels, the dashed line indicates the threshold 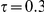; parameter values: system size is 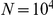, 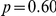, and 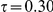.

More precisely, the above considerations are illustrated in Figure 7 for a lattice network. There we plot the distributions 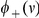 for different levels of connectivity 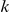 and parameter values 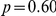 and 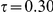 – recall that these values correspond to Phase I (with high turnover) in a *fully connected* network. Consider first the situation that arises for values of 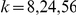 – i.e. low connectivity relative to the size of the system. Then we find that, among the nodes that are adopting action 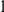, 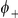 attributes a significant probability mass to those agents whose fraction of neighbors 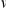 choosing action 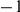 is below the threshold required to change (as marked by the vertical dashed line). Such nodes, therefore, will not change their action. And, as explained, this has the beneficial effect of limiting the extent of action turnover as compared with the global interaction setup. On the other hand, the inset of Figure 7 shows that, among the nodes that are adopting action 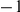, the distribution 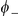 associates a large probability mass to those agents whose fraction of neighbors 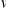 choosing the opposite action is above 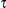. This ensures that there is a large enough flow from action 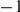 to action 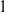. In conjunction, these two considerations lead to a situation that allows, first, for some limited nucleation around action 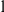 to take place, followed by the ensuing spread of this action across the whole system (Figure 7(*B*–*D*)).

Let us now reconsider the former line of reasoning when 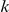 is large – in particular, take the case 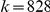 depicted in Figure 7. Then, the corresponding distribution 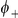 is highly concentrated around 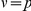, essentially all its probability mass associated to values that lie above 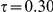. This means that the induced dynamics must be similar to that resulting from the complete-network setups, and thus too-fast turnover in action choice prevents the attainment of social learning. Clearly, social learning would also fail to occur for such high value of 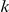 if the threshold 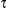 were large. In this case, however, the problem would be that the highly concentrated distributions 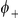 and 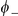 would have most of their probability mass lying below the threshold. This, in turn, would lead to the freezing of the initial conditions, which again is the behavior encountered for a complete network.

## Discussion

The paper has studied a simple model of social learning with the following features. Recurrently, agents receive an external (informative) signal on the relative merits of two actions. And, in that event, they switch to the action supported by the signal if, and only if, they find support for it among their peers - specifically, iff the fraction of these choosing that action lies above a certain threshold. Given the quality of the signal, correct social learning occurs iff the threshold is within some intermediate region, i.e. neither too high nor too low. For, if it is too high, the situation freezes at the configuration shaped at the beginning of the process; and if it is too low, the social dynamics enters into a process of continuous action turnover. A key conclusion is that social learning is a dichotomic phenomenon, i.e. it either occurs completely or not at all, depending on whether the threshold lies within or outside the aforementioned region.

These same qualitative conclusions are obtained, analytically, in the case of global interaction – which corresponds to a mean-field version of the model – as well as, numerically, in a wide range of social networks: complete graphs, regular lattices, Poisson random networks, and Barabási-Albert scale-free networks. However, the size of the parameter region where social learning occurs depends on the pattern of social interaction. In general, learning is enhanced (i.e. the size of the region enlarged) the less widespread is such interaction. This happens because genuinely local interaction favors a process of spatial nucleation and consolidation around the correct action, which can then spread to the whole population.

In sum, a central point that transpires from our work is that, in contrast to what most of the received socio-economic literature suggests, social learning is hardly a forgone conclusion. This, of course, is in line with the common wisdom that, paraphrasing a usual phrase, crowds are not always wise. In our threshold framework, this insight is robust to the topology or density of social interaction. Furthermore, our results highlight the importance of identifying the information diffusion mechanism, and the local sampling of the population provided by the social network. But future research should explore whether it is also robust to a number of important extensions. Just to mention a few, these should include (a) interagent heterogeneity – e.g. in their individual thresholds for change; (b) different behavioral rules – e.g. payoff-based imitation; or (c) the possibility that agents adjust their links, so that learning co-evolves with the social network.

## Methods

### Derivation of the threshold rule based on Bayesian update

Consider a population of 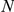 agents and let 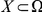 be the event which agents are interested in predicting, which is a subset of the sample space 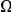.

The literature on Bayesian learning (see e.g. [24] and references therein) discusses cases where a population exchanges their prior information on 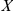, and the key quantity is the log-likelihood ratio:
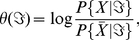
(3)


where 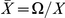 is the complement of 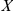 and 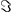 is a given information set. It is easy to show (see Ref. [24]) that when an agent with information 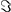 receives information 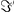 (e.g. because he/she exchanges information with another agent or receives a signal) the log-likelihood ratio changes in an additive manner
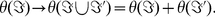
(4)


Such a dynamics, after an infinite number of updates, generically converge to a situation where 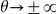 corresponding to the agent's beliefs converging to certainty on whether 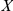 will occur or not. Naïvely speaking, Bayesian learning describes a dynamics under which individuals develop an “expertise” in the subject matter and converge to a state of absolute certainty about it.

This, however, seems implausible in cases where the subject matter is extremely complex (e.g. climate change). Then individuals are unlikely to become experts, rather they might hold a particular opinion, and display a corresponding action, to which they attach a given confidence. In this situation, if an agent receives enough signals contrary to his/her current action (or opinion) and enough of his/her neighbors also support the opposite view, then that agent may change opinion. Social learning then refers to the fact that the population as a whole may converge to a consensus, even though individuals themselves have beliefs which do not converge to certainty.

In order to model this setup, we assume from the outset that agents attach a certain confidence to their opinion, to that of their neighbors and to the signals they receive. Specifically, if 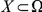 is the event which agents are interested in predicting, we assume that signals are random variables 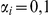 with
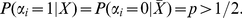
(5)


Agents, at any time, have their priors on 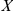 encoded in variables 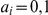, to which they attach a certain confidence 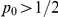, i.e.
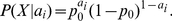
(6)


In words, 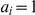 means that 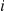 believes 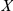 will occur and 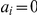 means he/she believes it will not, and 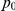 is the confidence they have in such a belief.

The variable 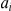 is observable by the nearest neighbors of 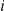. Reciprocally, 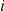 has access to the actions 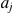 of his/her neighbors which reveal their opinions. We assume that agents have subjective beliefs on the correctness of the predictions of their peers, i.e.
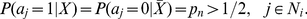
(7)


Here 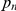 codifies the confidence which agents have on neighbors' opinions.

When news 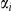 arrives, agent 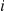 will recompute the posterior probability of 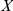, given the new evidence. At that time he/she will also look at neighbors and take their “opinion” into account. By Bayes rule:
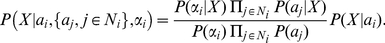
(8)


From this one can compute the likelihood ratio and conclude that if
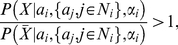
(9)


then agent 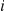 will update 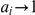. Otherwise he/she will set 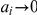. A simple calculation shows that, mathematically, this is equivalent to
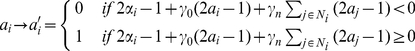
(10)


with
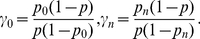
(11)


Now, the fact that agents change opinion only when they receive a signal which is opposite to their opinion (i.e. when 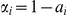) and not when their neighbors change opinion, implies that when 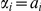 agents stick to their opinion. This requires
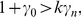
(12)


where 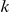 is the number of neighbors. On the other hand, when 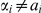, in order to change opinion, agent 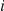 will need a fraction of supporters of the new opinion 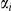 which is larger than
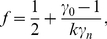
(13)


as it can easily be argued from Eq. (10).

This implies that, 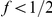 describes a situation where agents consider signals more informative than their own opinion (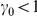, i.e. 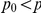) whereas in general they consider the opinion of others less reliable than signals and than their own (see Eq. 12). Indeed this analysis applies to the case where signals are nearly as informative as one's own opinion (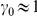) where 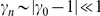.
